# Can real-time visual feedback during gait retraining reduce metabolic demand for individuals with transtibial amputation?

**DOI:** 10.1371/journal.pone.0171786

**Published:** 2017-02-09

**Authors:** Elizabeth Russell Esposito, Harmony S. Choi, Benjamin J. Darter, Jason M. Wilken

**Affiliations:** 1 Center for the Intrepid, Brooke Army Medical Center, JBSA, Ft. Sam Houston, Texas, United States of America; 2 Extremity Trauma and Amputation Center of Excellence, Ft. Sam Houston, Texas, United States of America; 3 Department of Physical Therapy, Virginia Commonwealth University, Richmond, Virginia, United States of America; University of Colorado Boulder, UNITED STATES

## Abstract

The metabolic demand of walking generally increases following lower extremity amputation. This study used real-time visual feedback to modify biomechanical factors linked to an elevated metabolic demand of walking in individuals with transtibial amputation. Eight persons with unilateral, traumatic transtibial amputation and 8 uninjured controls participated. Two separate bouts of real-time visual feedback were provided during a single session of gait retraining to reduce 1) center of mass sway and 2) thigh muscle activation magnitudes and duration. Baseline and post-intervention data were collected. Metabolic rate, heart rate, frontal plane center of mass sway, quadriceps and hamstrings muscle activity, and co-contraction indices were evaluated during steady state walking at a standardized speed. Visual feedback successfully decreased center of mass sway 12% (p = 0.006) and quadriceps activity 12% (p = 0.041); however, thigh muscle co-contraction indices were unchanged. Neither condition significantly affected metabolic rate during walking and heart rate increased with center-of-mass feedback. Metabolic rate, center of mass sway, and integrated quadriceps muscle activity were all not significantly different from controls. Attempts to modify gait to decrease metabolic demand may actually adversely increase the physiological effort of walking in individuals with lower extremity amputation who are young, active and approximate metabolic rates of able-bodied adults.

## Introduction

The metabolic demand of walking typically increases following limb loss. At similar speeds, individuals with a transtibial amputation (TTA) may expect metabolic rate to increase up to 9–33% [1–7]. Clinically, a greater metabolic effort contributes to slower walking velocities, a decline in the intensity or duration of activities, and ultimately the adoption a more sedentary lifestyle [8, 9]. Developing specific rehabilitation training interventions may help restore more normative energetic demands and potentially interrupt the progressive disablement that can occur after amputation.

Gait retraining strategies may be used to better optimize movement patterns in order to minimize the energetic demands of activity. Normal, or “optimized,” motions of the center of mass (COM), for example, are expected to result in the greatest walking economy [10, 11]. Excessive out-of-plane movements, such as side-to-side sway, can adversely affect the metabolic demand of walking [12, 13] and are commonly observed in individuals with amputations [14–16]. However, research suggests that reducing COM deviations by externally controlling frontal plane movement lowers the energy cost of walking [17]. Unfortunately, therapists are currently limited in their abilities to address these deviations using conventional rehabilitation methods. Offering gait retraining with real time visual feedback (VF) is one potential means of overcoming this limitation.

Real-time VF has been shown to enhance skill acquisition and retention, and may provide the opportunity to address deviations in a more direct manner [18, 19]. For example, Darter and Wilken [20] used therapist-directed training and real-time VF to improve frontal plane trunk, pelvis and hip motion, which decreased oxygen consumption 20% for an individual with a transfemoral amputation. Yang et al. [21] used real-time VF to improve stance time symmetry and found that it decreased trunk sway in two of the three patients who had a transtibial amputation. Davis et al. [22] investigated a single session of VF on push-off symmetry to lower the energy cost 6% in a group of individuals with TTA. Though these interventions did not provide feedback directly on COM motion, the authors utilized measures which undoubtedly influenced the path of the COM. Combined, the studies suggest that typical post-amputation gait compensations that result in asymmetries and excessive frontal plane motion are inefficient and contribute to increases in the metabolic demand of walking.

While asymmetries and COM control measures relate to metabolic demand, direct measures of muscle activity may provide greater insight into potential sources of excessive energy use. Persons with TTA often exhibit lower knee work and torque during stance, but have increased activation and a large co-contraction between the quadriceps and hamstring muscle groups [23, 24]. Co-contraction may result from efforts to stabilize the support limb and prevent falls as the quadriceps and hamstrings are co-active over 40% of the time during walking in individuals with amputations compared to 18% in non-amputees [23]. In early and mid-stance, vastus lateralis (VL) and biceps femoris (BF) co-contraction indices (CCI) may be as high as 95% in the residual limb compared to 62% in able-bodied controls [25]. In terminal swing, when the BF acts as the agonist, co-contraction can be almost twice as great in the residual limb as controls, but other research reports equivalent co-contraction levels in terminal swing [24]. These results, combined with an elevated metabolic demand in individuals with TTA, suggest that muscular co-contraction may add to the increased energy usage during walking, but further research is needed.

Reducing excessively high levels of muscle activation has been suggested as an approach for decreasing the energy cost, specifically by reducing the duration and magnitude of thigh muscle co-contraction, which is common in transtibial amputee gait [23]. Although muscles are the direct sites of energy consumption, no published study has implemented gait training focused on reducing co-contraction in persons with an amputation. It is, therefore, important to identify gait training parameters that reduce the energy cost of walking within a testing session prior to implementing them in large scale studies. Therefore, the purpose of this study was to determine if a single bout of real-time VF could reduce COM sway and muscular activity during walking. A secondary purpose of this study was to determine if these changes could reduce the metabolic demand of walking in individuals with traumatic transtibial amputation. We hypothesized that1) real time VF on COM sway and muscle activity would reduce the magnitudes of these measures during walking, 2) VF on direct measures of muscle activity would decrease metabolic demand more than COM control measures, and 3) changes in these biomechanical measures would reduce the metabolic demand of walking for individuals with unilateral TTA. The goal of assessing the efficacy of a single bout of VF was to determine if it had potential to be efficacious for a longer-term training intervention.

## Materials and methods

Eight male service members with unilateral TTA participated in this study. All participants were K4 level walkers (based on Medicare’s functional classification level) and characteristics, including age, height, mass and time in a prosthesis, can be found in Table 1. Exclusion criteria consisted of moderate to severe traumatic brain injury, involvement of the contralateral limb, medications known to influence metabolism, inability to walk on a treadmill without an assistive device and uncorrected vision impairment. Subjects wore a passive-dynamic, energy-storage-and-return prosthetic foot: Renegade (2) and Agilix (1) (Freedom Innovations, Irvine, CA, USA), Re-Flex Rotate (2) and Proprio Foot (1) (Össur, Reykjavik, Iceland), Pathfinder II (1) (Ohio Willow Wood, Mt. Sterling, OH, USA), and Soleus Tactical (1) (College Park Industries, Warren, MI, USA). Eight able-bodied control subjects also participated in the study. All subjects provided written informed consent in accordance with the procedures set forth by the approving institutional review board at Brooke Army Medical Center.

**Table 1:** Subject characteristics. Mean values (standard deviations), ranges and p-value are presented. Mass accounts for biological mass exclusive of a prosthesis.

	Age (years)	Height (m)	Mass (kg)	Speed (m/s)	Ambulation time (mo)
TTA	32.9 (5.7)	1.76 (0.08)	93.9 (20.3)	1.14 (0.11)	29 (38)
*range*	*25–42*	*1*.*66–1*.*88*	*57*.*0–118*.*8*	*0*.*97–1*.*25*	*3–120*
Control	29.4 (3.8)	1.80 (0.05)	80.4 (15.2)	1.17 (0.13)	
*range*	*27–43*	*1*.*74–1*.*88*	*52*.*8–104*.*0*	*0*.*94–1*.*27*	
p-value	0.169	0.210	0.154	0.670	

A domed Computer-Assisted Rehabilitation Environment (CAREN, Motek Medical, Netherlands) was used to deliver the real-time VF. Eight projectors displayed a 270 degree visual field in front of and around the subject for enhanced optical flow. Subjects walked on a 1.8 m wide by 2.8 m long treadmill in the center of the CAREN dome. The training environment consisted of a straight walking path through a forested area. Bipolar surface electrodes (1200 Hz, Motion Laboratory Systems, Inc. Baton Rouge, LA, USA) were placed bilaterally on the VL and BF muscles of the TTA group and on the right limb of the control subjects. Three maximal voluntary contractions (MVCs) were performed for each muscle and the largest value was used for EMG scaling purposes.

A twenty-four camera Vicon motion capture system (120 Hz Vicon, Inc., Oxford, UK) recorded three-dimensional trajectories of 57 markers placed on anatomical landmarks and body segments. A detailed description of the marker set and the full body model has been published previously [26].

Oxygen consumption was recorded using indirect calorimetry and a portable, telemetered metabolic unit (K4b2, Cosmed, Inc., Rome, Italy). A plastic mask was secured over the nose and mouth to capture all expired air and a heart rate (HR) monitor (Polar Electro Inc., Lake Success, New York) was worn about the chest.

All subjects walked in the CAREN at a standardized speed based on leg length, as measured from the greater trochanter to the floor of the unaffected limb (right limb for controls), and a dimensionless Froude number of 0.16 using the equation:

$$\text{Speed}=\;\sqrt{0.16\cdot \text{gravity }\cdot \text{leg length}}$$

[27]

The standardized speed facilitated equivalent task demand across individuals of different anthropometric characteristics (i.e. leg length) and the Froude number of 0.16 was selected because previous testing has shown this to be similar to a preferred walking speed for individuals with TTA. However, two TTA subjects requested to walk at slower velocities and, for consistency when comparing between groups, two control subjects (height matched within 5%) walked at equivalent Froude numbers. A safety harness that did not offer body weight support was worn to eliminate the risk of falling.

**Fig 1:**
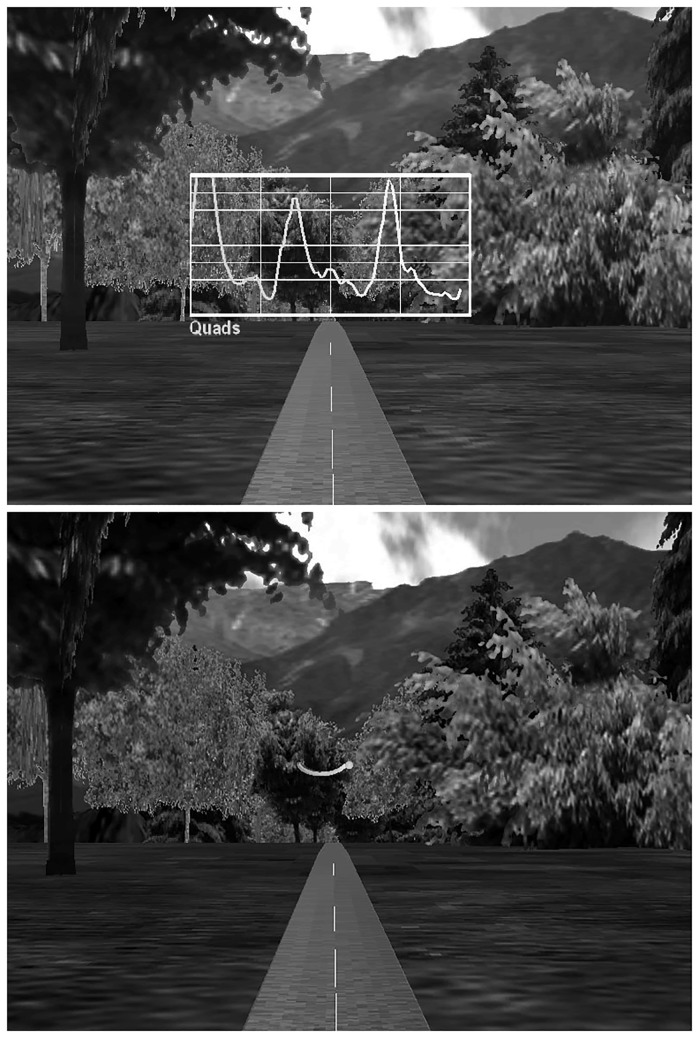
Visual display of example real-time feedback for EMG (top) and COM (bottom) within the Computer-Assisted Rehabilitation Environment (CAREN).

Prior to testing, subjects rested quietly in a seated position for 10 minutes while resting VO_2_ was collected. Resting VO_2_ was collected to determine a baseline level for subjects to return to between subsequent collections. Control subjects participated in a single bout of treadmill walking where no VF (NOF) was provided other than optic flow and TTA subjects participated in four bouts of treadmill walking with NOF washout sessions between the VF conditions (Table 2). During the NOF conditions, subjects were instructed to walk normally and only optic flow was provided. VF graphs were displayed directly in front of the subject (Fig 1).

**Table 2:** Testing conditions. The order of the conditions was not randomized. Each visual feedback (VF) condition was compared to the no feedback (NOF) condition preceding it.

	Rest	No Feedback (NOF)	Rest	EMG Feedback (VF_EMG_)	Rest	No Feedback (NOF)	Rest	COM Feedback (VF_COM_)
Control	X	X						
TTA	X	X	X	X	X	X	X	X

EMG VF (VF_EMG_) was provided from the residual limb VL. A visual trace of the rectified and smoothed (3 Hz low-pass filter) EMG data was displayed on a grid and subjects were instructed to decrease the peak and the duration of the quadriceps activity near heel strike using the grid as a reference. Verbally, subjects were instructed to make modifications such as “relaxing” the thigh muscles. During COM VF (VF_COM_) participants viewed a two-dimensional projection of the vertical and medial-lateral position of the seventh cervical marker and were instructed to increase symmetry and decrease marker excursion. The seventh cervical vertebrae marker was selected on the basis that trunk movement contributes largely to frontal plane COM motion and visualizing this marker has previously been a successful way to change frontal plane mechanics [20]. A physical therapist subjectively assessed the gait modifications by viewing the VF and provided verbal guidance in line with clinical gait retraining recommendations (e.g. “stand a little taller”, “relax your hip muscles”). A maximum time of 4 minutes was given to practice each VF task with guidance from the physical therapist, then subjects walked with only VF for approximately eight minutes or until physiological steady state VO_2_ was achieved for at least two minutes. Steady state was determined as a change of less than 10% in VO_2_ values during the final two minutes of data collection [28]. If steady state had not been achieved, subjects continued to walk until a two-minute plateau in oxygen consumption was reached. Verbal feedback from the therapist was not provided during these final minutes. Marker and EMG data were collected during the final 30 seconds of each steady state condition. Seated rest was provided between walking bouts to allow VO_2_ to return to baseline levels for at least two minutes.

Gross VO_2_ (scaled to biological mass) and HR data were averaged during the final two minutes of steady state data collection. COM and EMG data were initially analyzed in Visual3D (C-Motion, Germantown, MD). Marker data were smoothed at 6Hz using a fourth order Butterworth low-pass filter. A 13-segment, full body model [29] was created and used to calculate peak left and right frontal plane COM motion during ten consecutive strides. EMG data were filtered using a 20 Hz high-pass and 400 Hz low-pass Butterworth filter, then a moving RMS was performed using a 50ms window. In Matlab (The Mathworks, Inc., Natick, MA), EMG data were scaled to the peak MVC, normalized to 101 points, and peak values for the VL and BF were analyzed between 0–20% and 80–100% of the gait cycle (starting at heel strike), respectively. Integrated VL (iVL) and BF (iBF) activity were used in CCI calculations with a modified version of Falconer and Winter’s method [30] that used the VL as the agonist (BF as antagonist) during the first 20% of stance, the BF as the agonist (VL as antagonist) during the last 20% [25] and the equation:

$$\text{CCI}=100\%\;\times \;\frac{2\cdot \;ʃ\text{antagonist}}{ʃ\;\left(\text{agonist}+\text{antagonist}\right)}$$

Each VF condition was compared to the preceding NOF conditions. A two-way repeated measures analysis of variance assessed main effects of limb and VF condition for all EMG conditions within the patient group. Paired t-tests assessed differences in COM sway, VO_2_ and HR and also percent change from NOF between VF_EMG_ and VF_COM_. Comparisons to controls were performed for COM sway, EMG measures, VO_2_, and HR using a one-way ANOVA with Dunnet’s post-hoc tests. The criterion for statistical significance was set at p<0.05. Effect sizes (d) were calculated between VF and NOF conditions within the TTA group and 0.2, 0.5 and 0.8 denoted small, moderate, and large effects, respectively [31].

## Results

**Fig 2:**
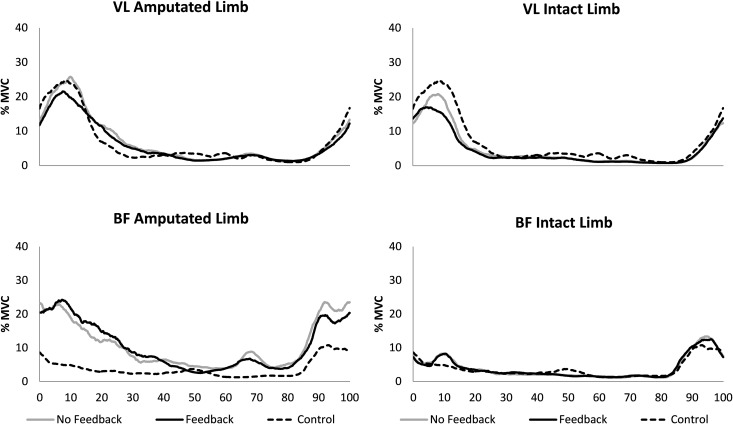
Ensemble averaged EMG data from the VL and BF muscles normalized to the gait cycle.

**Fig 3:**
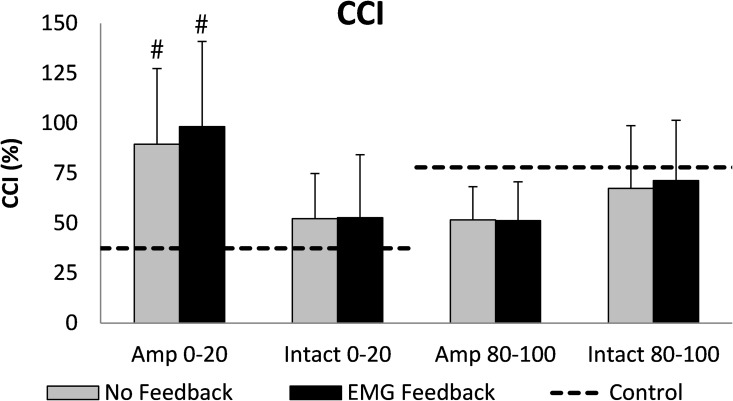
Mean (SD bars) CCI for the thigh muscles during the first 20% and last 20% of the gait cycle. During the first 20%, the VL acts as an agonist (BF antagonist) and during the last 20% the BF acts as an agonist (VL antagonist) [25]. # indicates a significant difference from the control value; there were no significant differences between feedback conditions.

There were no significant interactions between condition and limb for any of the EMG measures (Fig 2). In the residual limb, VF_EMG_ significantly reduced iVL activity during early stance (p = 0.041, d = 0.339) but did not affect iBF activity (p = 0.548, d = 0.143). Despite an average 13.3% decrease in the peak VL EMG activity during the first 20% of the gait cycle with VF_EMG_, this change did not reach statistical significance (p = 0.051, d = 0.436). During terminal swing, VF_EMG_ did not significantly decrease peak BF (p = 0.566, d = 0.121), iBF (p = 0.302, d = 0.311) or iVL (p = 0.453, d = 0.352) EMG activity. CCI were not significantly different between VF conditions (early stance: p = 0.479, d = 0.217, terminal swing: p = 0.699, d = 0.020, Fig 3).

There were significant differences between limbs such that iBF activity was significantly greater in the amputated limb in both early stance (p = 0.036) and terminal swing (p = 0.011), but neither peak (p = 0.162) nor iVL activity was different between limbs (early stance: p = 0.133, terminal swing: p = 0.506). CCI were significantly greater in the amputated limb than intact in early stance (p = 0.047) but were significantly lower in the amputated limb during terminal swing (p = 0.036).

Compared to controls, there were no significant differences in peak or iVL activity in any of the conditions. iBF was, however, significantly greater than controls on the amputated side in early stance (NOF: p = 0.022, VF_EMG_: p = 0.008); in terminal swing iBF was greater only in the NOF condition (NOF: p = 0.008, VF_EMG_: p = 0.068) While the VF did not change BF EMG during the last 20% of the gait cycle (p = 0.201), values were significantly greater for the TTA group than controls across both conditions (NOF_EMG_: p = 0.006, VF_EMG_: p = 0.009).

**Fig 4:**
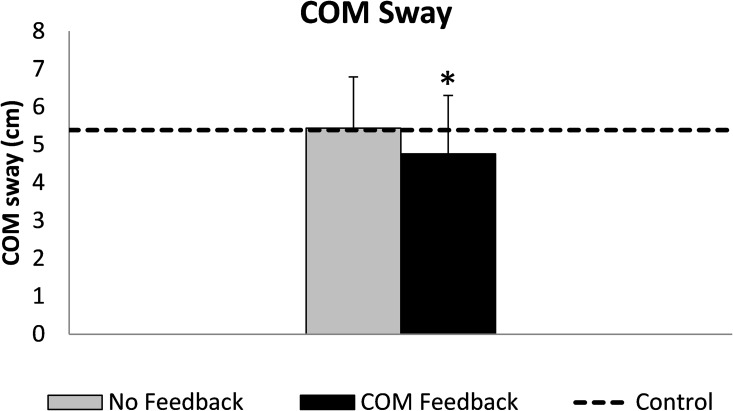
Mean (SD bars) maximal frontal plane COM sway during each stride. The horizontal dashed line represents the mean value from the control data when walking with no feedback. * indicates a significant difference from the no feedback condition; there were no significant differences from controls.

Study participants were able to significantly decrease their frontal plane COM sway 12.5% using VF_COM_ (p = 0.006, d = 0.467, Fig 4). However, during both the NOF_COM_ and VF_COM_ conditions, peak frontal plane COM sway was not significantly different from controls (NOF_COM_: p = 0.980, VF_COM_: p = 0.558).

**Fig 5:**
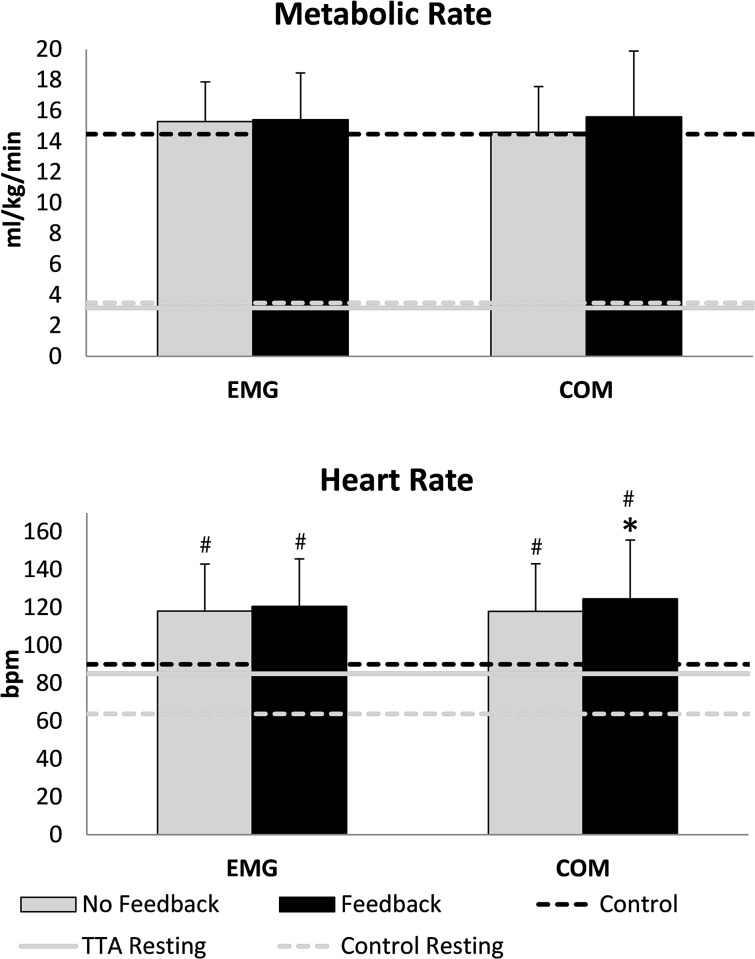
Mean (SD bars) metabolic (top) and heart rate (bottom). The black horizontal dashed line represents the mean value from the control data when walking with no feedback. Gray horizontal lines represent resting values for each subject group (dashed—control, solid—TTA). # indicates a significant difference from the control value and * indicates a significant difference from the no feedback condition. Metabolic rate was scaled to biological body mass.

VO_2_ did not change during either VF condition (VF_EMG_: p = 0.796, d = 0.044, VF_COM_: p = 0.796, d = 0.274). Metabolic cost values also did not change, as speeds did not differ between groups, and values for the TTA group were NOF_EMG_: 0.22 (0.04), VF_EMG_: 0.22 (0.04), NOF_COM_: 0.21 (0.04) VF_COM_: 0.23 (0.06) ml/kg/m and values for the control group were 0.21 (0.03) ml/kg/m. HR was unaffected by VF_EMG_ (p = 0.152, d = 0.101) but VF_COM_ resulted in a small increase in HR (VF_COM_: p = 0.039) (Fig 5). VO_2_ in the TTA group was not significantly different from able-bodied individuals in any condition (NOF_EMG_: p = 0.725, NOF_COM_: p = 0.996, VF_EMG_: p = 0.655, VF_COM_: p = 0.689) but HR was significantly greater across all conditions (NOF_EMG_: p = 0.007, NOF_COM_: p = 0.017, VF_EMG_: p = 0.004, VF_COM_: p = 0.005). Ambulation time did not affect VO_2_ as values during the two NOF conditions were not correlated with ambulation time (average R^2^ = -0.111).

## Discussion

The purpose of this study was to determine if a single bout of real-time VF on COM sway and muscular activity could reduce the magnitudes of these variables and also reduce the metabolic demand of walking in individuals with traumatic transtibial amputations. Since muscular contraction requires metabolic energy, it was expected that direct feedback aimed at reducing quadriceps-hamstrings co-contraction could reduce the cost of walking. It was also expected that reducing frontal plane sway would reduce muscular efforts needed to control these deviations and, thus, reduce the metabolic cost of walking. The use of a single bout of real-time VF successfully reduced quadriceps activation in early stance and frontal plane COM sway in individuals with unilateral TTA, however, these changes did not coincide with a change in VO_2_ or HR and effect sizes were generally small. Since VO_2_ was not different from controls during the NOF conditions we expect there was little room for improvement in the cost of walking. These findings contradict the larger body of literature on individuals with unilateral lower limb amputations, who typically have a 9–33% greater metabolic demand than able-bodied individuals [1–7], but agree with recent findings on a similar patient population of young, active individuals with TTA [32].

Recently, Russell Esposito et al. [32] also found that individuals with traumatic unilateral TTA who were young and fit, relative to the general population of amputees, had equivalent metabolic rates to able-bodied individuals when walking at equivalent speeds. In further agreement with the present study, HR was greater in the TTA group. One additional study on running found that athletes with TTA were also able to achieve similar metabolic rates as able-bodied controls, albeit greater HR, when running at the same speed in a running-specific prosthesis [33]. The results of the present study, combined with the limited available literature, indicate that when the patient is relatively young, active and highly motivated, interventions designed to decrease energy expenditure may not always be necessary. However, these gait retraining interventions may still be beneficial for relatively older and less active individuals for whom walking may be more metabolically costly.

The gait retraining interventions successfully reduced iVL EMG and COM sway with no adverse increase in VO_2_. The changes elicited and the effect sizes were, however, relatively small. COM sway, for example, decreased less than a centimeter and baseline values from the NOF condition were not greater than controls. Other interventions have also sought to control frontal plane COM sway in individuals with amputation. Mechanically induced lateral stabilization has been used to decrease COM sway in individuals with TTA, but the effect on energy expenditure was small [17]. Because lateral stabilization and COM sway are important components of balance control, and given other published literature in this population [34, 35], it is likely that individuals with TTA in the current study did not have problems with balance control that were sufficiently large to affect the metabolic cost of walking on level ground. Therefore, the authors can conclude that reducing COM sway is not a necessary adaptation for individuals with TTA if the metabolic demands for walking are equivalent to able-bodied individuals.

Contrary to our second hypothesis, direct feedback of muscle activity did not have a greater effect on VO2 than feedback on COM sway. Although this study focused on providing VF_EMG_ on the VL (with verbal feedback on the thigh muscles, in general), these efforts to specifically reduce quadriceps activation did not impact the hamstrings measures on the amputated limb were elevated to a greater extent than controls and the intact limb. Previous studies have suggested that this greater activation in early stance helps stabilize the knee joint [24, 36] and/or increase the amputated limb’s propulsive capabilities [37, 38]. The BF typically reach peak activation in terminal swing as they eccentrically act to decelerate the lower leg [39]. Similar to previous research on amputees [24], the peak BF activity occurred between terminal swing or just following heel strike in the TTA group, whereas peak activity always occurred in terminal swing in controls. The consequence of elevated BF activity during stance was greater CCI in the amputated limb in early stance. Surprisingly, however, the excessive muscular action could not be attributed to a greater metabolic demand of activity, potentially because the lacking ankle musculature incurs no metabolic penalty, the overall mass of the limb is reduced, potentially reducing the cost of swing, and the energy-storing-and-returning prosthetic device provides some of the work required for walking without requiring metabolic energy.

Although individuals with TTA in this study were not able to further reduce their metabolic cost of walking below that of able-bodied controls, this treatment strategy was able to elicit gait modifications which may benefit other populations. For example, using real-time VF on trunk motion or select spine muscles (e.g. [40]) could potentially reduce certain gait deviations related to low back pain. In addition, individuals with transfemoral amputations have a 30–60% greater metabolic cost of walking than their able-bodied counterparts [5, 9, 41–46]. They also experience greater frontal plane trunk lean [47] and quadriceps-hamstrings co-contraction [48] during walking than non-amputees. If real-time VF can decrease the trunk sway and/or excessive muscle activity, it may have the potential to reduce the metabolic cost in individuals with higher levels of amputation. A longer-term training intervention may be useful for improving gait and reducing the metabolic cost of walking (e.g. [49]) but further study would be needed to determine its efficacy for this population.

### Study limitations

The primary limitation of this study is that feedback was provided based on the assumptions that 1) the metabolic cost of locomotion would be elevated in this population and 2) metabolic demand may be reduced if walking biomechanics could approximate those of able-bodied individuals. Subjects were not screened for elevated metabolic rates or excessive COM sway and muscle activity prior to study enrollment. It was expected based on previous literature that these deviations would be present in most or all of the population of individuals with unilateral TTA who participated in this study, but only CCI in early stance was elevated relative to controls. An additional limitation is that muscle activations were scaled to MVC values and full recruitment of motor units may have been difficult to achieve for an amputee population [50] or affected by residual limb pain [51]. However, the use of MVCs was considered optimal over other methods [52] and the primary focus of this study was the within-subjects effect of the real time VF, which would be unaffected by the scaling method.

## Conclusions

The purpose of this study was to explore the use of a single bout of real-time VF to attenuate excessive quadriceps-hamstrings activity and co-activation and COM sway to reduce the metabolic demand of walking. A single bout of real-time VF during walking was an effective way to reduce both COM sway and quadriceps activation in individuals with TTA. However, these changes in biomechanics did not reduce the metabolic demand of walking. In addition, feedback on muscle activity was not more effective than proxy measures of COM control. The lack of differences in VO_2_ between TTA and controls may be attributable to the young, very active and highly motivated participants in this study. Therefore, attempts to decrease VO_2_ through gait training likely benefit only those with increased metabolic demands relative to healthy able-bodied adults.

## Supporting information

S1 File Supporting data from the individual subjects included in this manuscript. (XLSX)

## Abbreviations

BF: biceps femoris
CAREN: computer assisted rehabilitation environment
CCI: co-contraction index
COM: center of mass
EMG: electromyography
iBF: integrated biceps femoris electromyography
iVL: integrated vastus lateralis electromyography
MVC: maximal voluntary contraction
NOF: no feedback
NOF_COM_: no feedback of center of mass sway
NOF_EMG_: no feedback of electromyography
RMS: root mean square
TTA: transtibial amputation
VF: visual feedback
VF_COM_: visual feedback of center of mass sway
VF_EMG_: visual feedback of electromyography
VL: vastus lateralis
VO_2_: metabolic rate
